# Relationship between bronchial asthma and COVID-19 infection in adults: clinical and laboratory assessment

**DOI:** 10.1186/s43168-023-00183-9

**Published:** 2023-02-20

**Authors:** Abeer M. Rawy, Mohamed S. Sadek, Mysara M. Mogahed, Afaf Khamis, Amira H. Allam

**Affiliations:** 1 Department of Chest, Faculty of Medicine, Benha University, Benha, 13512 Egypt; 2 Department of Internal Medicine, Faculty of Medicine, Benha University, Benha, Egypt; 3 Department of Clinical Pathology, Faculty of Medicine, Benha University, Benha, Egypt

**Keywords:** COVID-19, Asthma, Eosinophils

## Abstract

**Background:**

Asthma is still considered a major chronic respiratory disease that affects a large number in the world. The association between COVID-19 infection and asthma was studied in different ways focusing on hospital-admitted patients. This study aimed to assess the outcome of patients with asthma and/or COVID infection in adults attending outpatient pulmonary clinic over three successive months from clinical and laboratory point of view.

**Patients and methods:**

The current study was a retrospective observational study involving 898 patients attending the outpatient pulmonary clinic of a Saudi Arabian private hospital over three successive months from the 1st of December 2020 to the end of February 2021. Patients were divided into three groups: group 1—COVID-19 infected with asthma (312); group 2—COVID-19 infected with no asthma (286); and group 3—COVID-19 non-infected with asthma (300).

**Results and conclusions:**

Results showed the best patient’s outcome was seen in asthmatic patients without COVID-19 infection followed by asthmatic patient with COVID-19 infection. There was a significant statistical difference in eosinophil count between COVID-19-infected patients with asthma and COVID-19 infected without asthma. Also, it was shown that the most common cause of hospitalization in asthmatic patients with COVID-19 infection was pneumonia followed by gastroenteritis and not an asthma exacerbation.

## Introduction

Asthma is considered an airway complex inflammatory disorder that is classified into eosinophilic and non-eosinophilic phenotypes [1]. The majority of the patients with asthma demonstrate a predominantly T helper type 2 (Th2) immune response, in which the type 2 immune cells are playing a major role in asthma pathogenesis. In a relatively lower percentage of asthma patients, the T helper type 17 (Th17) endotype is associated with a predominantly neutrophilic inflammation with different pathophysiological mechanisms [2].

The COVID-19 infection is spreading at an alarming rate with over 116 million confirmed cases, including 2.5 million deaths, globally as of 9 March 2021 [2]. Patients with chronic respiratory diseases such as asthma would be expected to be at high risk of COVID-19 as the virus primarily targets the airways and lung parenchyma [3].

Currently, the prevalence of asthma among patients with COVID-19 is controversial. This dispute could be explained by the prevalence of asthma phenotypes in the general population of a specific area and the COVID-19 outbreak impact in that area [4]. Immunoregulation factors, as Th2- and Th17-driven inflammation, are likely to modify the risk of COVID-19 outcomes in asthma. The hallmark characteristics of bronchial asthma, as eosinophilia and Th2 inflammation, are potentially capable of promoting viral clearance and inducing antiviral immunity, which may therefore account for the low prevalence of asthma reported among COVID-19 individuals in some studies [5]. Low eosinophil count is expected to be a clinical characteristic of acute respiratory deterioration in patients with COVID-19 [6]. The increase in eosinophil count may serve as an indicator of COVID-19 improvement [7]. This study aimed to assess the outcome of patients with asthma and/or COVID infection in adults attending outpatient pulmonary clinic over three successive months from clinical and laboratory point of view.

### Patients and methods

**Fig. 1:**
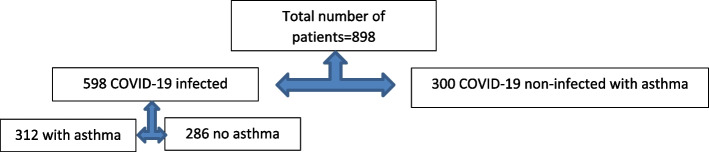
Number of cases enrolled in the study

The current study is a retrospective analytical study on 898 patients attending a pulmonary clinic in a Saudi Arabian private hospital over three successive months starting from the 1st of December 2020 to the end of February 2021. Patients were classified according to previous history of asthma and/or infection with COVID-19 in the previous 6 months. Patients were divided into two main groups according to the history of COVID-19 infection. Each group was divided into asthma and non-asthma groups. Patients who had no history of asthma and had no history of previous infection with COVID-19 were excluded from the study. So, we get three groups: group 1—COVID-19 positive with asthma; group 2—COVID-19 positive with no asthma; and group 3—COVID-19 negative with asthma (Fig. 1). The study was approved by the local internal ethics committee and patient’s acceptance to reveal their data was received prior to the study.

## Methods

Data were collected from medical records including the history of COVID-19 infection, presenting symptoms and clinical examination, and hospitalization either ICU or ward. For asthma patients, their full data were collected regarding asthma control in the last 3 months following GINA criteria for asthma control [8]. Investigations included CBC with differential count, WBC, lymphocytes, eosinophils, Hgb and platelets, inflammatory markers as D-dimer, LDH and ferritin level, electrolytes, BUN, and serum creatinine.

Diagnosis of COVID-19 infection was made by a positive nasopharyngeal and throat swabs COVID-19 polymerase chain reaction (PCR). The data were collected from patients’ medical records including medical history, demographic information such as age, gender, symptoms of COVID-19, time of onset of symptoms, the physical examination at admission, during hospitalization, medications prescribed for COIVID-19 treatment, and laboratory examinations which were performed for all groups.

The data were analyzed using SPSS 22 (SPSS Inc., Chicago, IL, USA). Parametric data were used. The results were presented as percentile (absolute numbers): mean and standard deviation. Quantitative data were presented as median (interquartile range) (IQR, presented as first quartile–third quartile). Qualitative data were expressed as percentage (%) [9]. Non-paired *T* test was used to compare 2 groups of data.

## Results

The current study included 1309 patients who attended the outpatient pulmonary clinic in three successive months. The results in Table 1 show highly significant statistical difference between both groups (1 and 2) regarding age, smoking index, and duration of hospital stay. The mean age was higher in group 1 while the smoking index and duration of hospital stay were higher in group 2. Patients’ vital signs revealed oxygen saturation higher in group 1 while temperature, heart rate, and respiratory rate were higher in group 2 with highly significant statistical differences. As regards patients’ laboratory findings, there was no significant difference in the total leucocytic count and lymphocytic percentage between both groups while there were highly significant statistical differences in eosinophilic percentage, Hgb, platelet count, and renal functions. There was a significant statistical difference between both groups (1 and 2) as regards Na, K, Ca, D-dimer, and ferritin level while no statistical differences as regards Mg and LDH.

**Table 1:** Demographic, clinical, and laboratory data of the study groups

	**Group 1**	**Group2**^a^	***P* value**	**Group 3**		***P* value**
Number	312 (52.2%)	286 (47.8%)		300 (45.45%)		
Gender						
Males	273 (87.5%)	169 (59.1%)		180 (60%)		
Females	39 (12.5%)	117 (40.9%)		120 (40%)		
Age	45.5 ± 12.6	40.4 ± 12.9	< .0001	40.2 ± 13.3		
Smoking status						
Smokers	182 (58.3%)	130 (45.5%)		144 (48%)		
Nonsmokers	130 (41.7%)	156 (54.5%)		156 (52%)		
Smoking index	222.8 ± 118	321 ± 145.9	< .0001	239 ± 127		
Hospital stay in days	23.6 ± 14.8	29.7 ± 16.2		7.3 ± 4.5		
Vital signs						
SpO2%	95.4 ± 2.8	93.8 ± 6.4	< .0001	97.4 ± 1.3		< 0.0001
Temp (°C)	37.4 ± 0.73	38.01 ± 0.92	< .0001	36.7 ± 0.31		< 0.0001
Pulse rate (beat/min)	93.13 ± 18.6	96.25 ± 9.26	.0022	78.3 ± 11.7		< 0.0001
Respiratory rate (cycle/min)	19.7 ± 2.58	20.4 ± 3.5	.0027	19.37 ± 1.7		< 0.0001
Blood pressure (mmHg)						
Systolic	133.5 ± 26.3	120.1 ± 10.3	< .0001	120.4 ± 13.3		< 0.0001
Diastolic	83.5 ± 11.5	76.2 ± 8.3	< .0001	79.6 ± 11.02		< 0.0001
Laboratory						
WBC (10^3^/μL)	6.78 ± 2.13	6.8 ± 5.39	.9481	7.1 ± 1.8		= 0.0395
Lymphocytes (10^3^/μL)	22.2 ± 8.3	22.7 ± 8.4	.4647	31.5 ± 6.8		< 0.0001
Eosinophils (10^3^/μL)	5.3 ± 5.5	0.735 ± 0.857	< .0001	9.5 ± 4.97		< 0.0001
Hgb (mg/dl)	14.95 ± 1.32	13.5 ± 1.66	< .0001	15.364 ± 1.18		< 0.0001
Platelets (10^3^/μL)	257.5 ± 60.4	192.7 ± 62.9	< .0001	228.24 ± 65.6		< 0.0001
S. creatinine (mg/dl)	0.98 ± 0.18	1.15 ± 0.75	< .0001	0.96 ± 0.2		.1771
BUN (mg/dl)	11.04 ± 3.3	13.7 ± 10.4	< .0001	11.9 ± 4.13		.0031
Electrolytes						
Na (mmol/L)	139.5 ± 3.2	138.3 ± 4.7	< .0001		138.56 ± 4.6	.0021
K (mmol/L)	4.05 ± 0.24	3.9 ± 0.4	< .0001		4.03 ± 0.48	.4883
Mg (mg/dl)	2.035 ± 0.15	2.04 ± 0.27	.7613		1.98 ± 0.19	< .0001
Ca (mg/dl)	8.7 ± 0.53	9.1 ± 0.62	< .0001		8.2 ± 1.06	< .0001
D-Dimer (μg/ml)	0.86 ± 0.54	1.08 ± 1.4	.0058		0.47 ± 0.27	< .0001
LDH (U/L)	357.2 ± 153.2	340 ± 176	.1705		198.6 ± 46.7	< .0001
Ferritin level (μg/L)	506.5 ± 504.6	373.7 ± 306	< .0001		134.5 ± 78.2	< .0001

*WBC* White blood cells, *Hgb* Hemoglobin, *S creatinine* Serum creatinine, *BUN* Blood urea nitrogen, *LDH* Lactate dehydrogenase

^a^Comparison between group 1 and group 2; group 1 and group 3 was done separately

Table 1 shows a highly significant statistical difference between groups 1 and 3 regarding mean age and duration of hospital stay; both were higher in group 1. There was no significant statistical difference in smoking index. Regarding patients’ vital signs, oxygen saturation was higher in group 3 while temperature, heart rate, respiratory rate, and systolic and diastolic blood pressures were higher in group 1 with highly significant differences. Patients’ laboratory findings (Table 1) showed a significant statistical difference in total leucocytic count between both groups. Lymphocytes and eosinophils were lower in group 1 than group 3 with a highly significant difference. In addition, there were highly significant statistical differences in D-dimer, LDH, and ferritin levels between groups 1 and 3 with relatively higher values in group 1.

The presenting symptoms of patients and any co-morbidities are presented in Table 2. The most common presenting symptoms in group 1 were cough (95.8%) followed by fever (75%), dyspnea (58.3%), and body aches (48.7%). In group 2, the most common presenting symptoms were fever (86.4%) followed by cough (68.2%), dyspnea (40.9%), upper respiratory tract symptoms (URT) (45.5%), and general body aches (40.9%). In group 3, the most common presenting symptoms were dyspnea on the top (96.3%) followed by cough (67%), general body aches (71.7%), headache (22.3%), fever (16.3%), and URT symptoms. The most common associated co-morbidities were HTN which is highest in group 2 (26.2%), followed by group 3 (21%), then group 1 (12.5%). Diabetes mellitus is the second common associated co-morbidity, with 18%, 12.9%, and 8.7% in group 3, group 2, and group 1, respectively.

**Table 2:** Presenting symptoms and co-morbidities

	G 1: Covid-19 infected with asthma 312	G 2: Covid-19 infected with no asthma 286	G 3: Covid-19 non-infected with asthma 300
Symptoms			
Fever	234 (75%)	247 (86.4%)	49 (16.3%)
Cough	299 (95.8%)	195 (68.2%)	201 (67%)
Dyspnea	182 (58.3%)	117 (40.9%)	289 (96.3%)
Headache	65 (20.8%)	39 (13.6%)	67 (22.3%)
Loss of smell and taste	11 (3.5%)	13 (4.5%)	0 (0%)
General body ache	152 (48.7%)	117 (40.9%)	215 (71.7%)
URT symptoms	26 (8.3%)	130 (45.5%)	48 (16%)
GIT symptoms:	39 (12.5%)	39 (13.6%)	23 (7.7%)
Co-morbidities			
DM	27 (8.7%)	37 (12.9%)	54 (18%)
HTN	39 (12.5%)	75 (26.2%)	63 (21%)
CKD	14 (4.5%)	21 (7.3%)	9 (3%)
Neurological	13 (4.1%)	4 (1.4%)	13 (4.3%)
Allergic rhinitis	29 (9.3%)	17 (5.9%)	86 (28.7%)

*URT* Upper respiratory tract, *GIT* Gastrointestinal tract, *DM* Diabetes mellitus, *HTN* Hypertension, *CKD* Chronic kidney disease

**Fig. 2:**
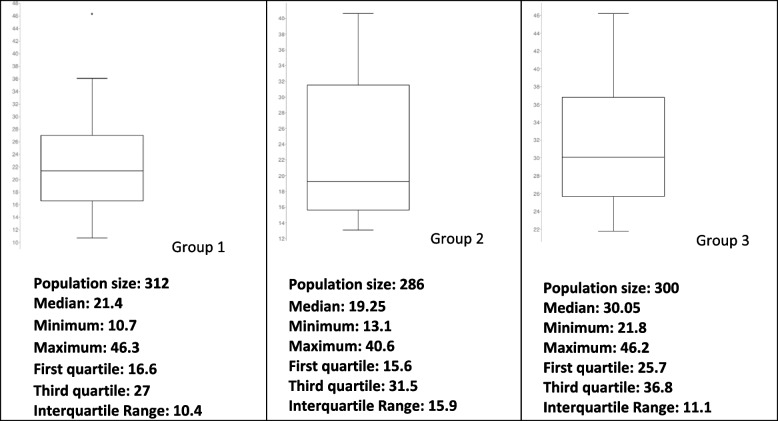
Eosinophils % interquartile range

**Fig. 3:**
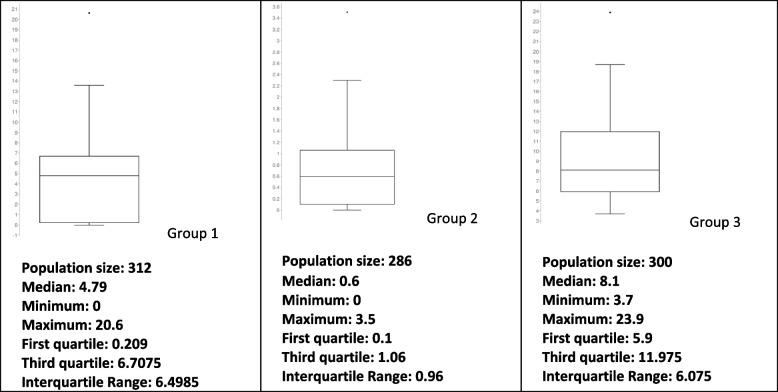
Lymphocytes % interquartile range

The eosinophil and lymphocytic percentages are presented in Figs. 2 and 3. Eosinopenia and lymphopenia were features in COVID-19-infected groups.

The hospital and home management are shown in Table 3. The highest rate of hospital admission was seen in group 2. There were 161 patients (56.3%) need hospital management with 33.9% in the ward while 22.4% in ICU. The need for mechanical ventilation is indicated in 3.5% of the patient. The other groups (1 and 3) showed lesser percentage of hospital admission in either ward or ICU. There was no significant difference in asthma control in the 3 months prior to the study between asthmatic patients with or without COVID in the preceding 6 months (Table 4).

**Table 3:** Patients place of management

**Group**	**Hospital admission**			**Non-hospital admission**
	**Ward admission**	**ICU admission**		
		**Need MV**	**No MV**	
Group 1	73(23.4%)	7 (2.2%)	19 (6.1%)	213(68.3%)
Group 2	97 (33.9%)	10 (3.5%)	54 (18.9%)	125 (43.7%)
Group 3	63 (21%)	3 (1%)	16 (5.3%)	218 (72.7%)

**Table 4:** Asthma medications and patient’s control in the last 3 months

Medications	G 1: Covid-19 infected with asthma (312)	G 3: Covid-19 non-infected with asthma (300)	Remarks
ICS	9 (2.9%)	24 (8%)	
ICS + LABA	303 (97%)	276 (92%)	
Inhaled LAMA	43 (13.8%)	32 (10.7%)	
Leukotriene’s modifier	249 (79.8%)	218 (72.7%)	
OCS	23 (7.4%)	5 (1.7%)	
Biological treatment	4 (1%)	1 (0.33%)	
Fully controlled	183 (58.6%)	180 (60%)	
Partially controlled	118 (37.8%)	113 (37.7%)	
Non-controlled	11 (3.5%)	7 (2.3%)	

*ICS* Inhaled corticosteroids, *LABA* Long acting B2 agonists, *LAMA* Long acting muscarinic antagonists, *OCT* Oral corticosteroid

## Discussion

This study aims to assess the outcome in patients with asthma and/or COVID 19 infection in adults attending outpatient pulmonary clinic over 3 months from clinical and laboratory point of view. The study included 898 patients with a history of asthma and/or history of COVID-19 infection after exclusion of 411 non-asthmatic non-COVID-19-infected patients. The patients were classified into 3 main groups: group 1—COVID-19 infected with asthma (312 patients); group 2—COVID-19 infected with no asthma (286 patients); and group 3: COVID-19 non-infected with asthma (300).

The best patient’s outcome was seen in group 3 asthmatic patients without COVID-19 infection followed by group 1 asthmatic patients with COVID-19 infection. The outcome was measured according to severity of symptoms, days of hospital admission in either ICU or ward admission, and the need for mechanical ventilation. The majority of patients with asthma attacks were treated at home in both COVID-19- and non-COVID-19-infected groups. These results were in agreement with the review done by Richard et al. [10] which showed that asthma prevalence among those hospitalized with COVID-19 is similar to those general population with asthma.

Group 2 which represents patients with a history of COVID-19 infection without previous history of asthma showed higher percentage of hospitalization, ICU admission, and longer duration of hospital stay than the other two groups (Table 3). While analyzing patients’ data in group 1, we found that the severe cases of asthma with COVID-19 infection that needed hospital admission were having other co-morbidities as diabetes mellitus, hypertension, or chronic kidney disease (Table 3). The explanation could be the increased distribution of the angiotensin-converting enzyme receptor (ACE2) in the respiratory airway epithelium in those co-morbid patients [11, 12]. On the other hand, the inhaled corticosteroids cause depression to ACE2 expression in the respiratory epithelium which explains relatively better outcome in asthma patient on treatment with ICS as budesonide [13].

The pathobiological mechanisms which explain the protection in patients with asthma to catch severe COVID-19 infection are still for more research. But there was a suggestion that decreased ACE2 receptor expression may lower the risk of COVID-19 severity and mortality in patients with predominantly atopic asthma [12, 14]. Also, T-helper 2 (Th2) immune response in patients with asthma may counteract the inflammation induced by SARS-CoV-2 infection [15]. It was observed that there is a significantly difference in eosinophil count between COVID-19-infected patients with asthma and COVID-19 infected without asthma. The protective role of eosinophils against SARS COV-2 viral infection is related to the big specific granules in eosinophils, the major basic protein, eosinophil cationic protein, and eosinophil neurotoxin which had a role in protective immunity against viruses [16].

The current results showed the most common cause of hospitalization in asthmatic patients with COVID-19 infection was pneumonia followed by gastroenteritis and not an asthma exacerbation [17]. This finding was in agreement with Beurnier et al. who studied all admitted patient with COVID-19 infection and reported asthma history in his institution. He found none of those patients presented with an asthma exacerbation but they were admitted due to COVID-19 pneumonia [18].

The limitations of this study were the study was a retrospective and done in one medical center. In addition, we could not calculate the mortality rate as it was only concerned with the surviving patients after infection.

## Conclusion

COVID-19-infected patients with asthma showed better outcome, as measured by days of hospital admission in either ICU or ward admission and the need for mechanical ventilation, than COVID non-asthmatics. There was no difference in asthma control between both groups. The most common causes of hospitalization in asthmatic patients with COVID-19 infection were pneumonia followed by gastroenteritis and not an asthma exacerbation.

## Data Availability

The data that support the findings of this study are available from the corresponding author, AHA, upon reasonable request.
